# Mapping cardiac and respiratory pulsations simultaneously with functional connectivity in the rat brain using zero echo time fMRI

**DOI:** 10.1177/0271678X261445230

**Published:** 2026-05-06

**Authors:** Ekaterina Paasonen, Petteri Stenroos, Antonio Caulin Atienzar, Juhani Utriainen, Raimo A Salo, Mikko Kettunen, Sara Ponticorvo, Shalom Michaeli, Silvia Mangia, Jaakko Paasonen, Olli Gröhn

**Affiliations:** 1 AI Virtanen Institute for Molecular Sciences, University of Eastern Finland, Kuopio, Finland; 2 Neurocenter, Kuopio University Hospital, Kuopio, Finland; 3 Department of Bioengineering, University Carlos III of Madrid, Madrid, Spain; 4 Center for Magnetic Resonance Research, University of Minnesota, Minneapolis, MN, USA

**Keywords:** Zero echo time, rat, functional magnetic resonance imaging, vascular pulsations, brain

## Abstract

Vascular pulsations are increasingly recognized as key contributors to cerebral perfusion and brain clearance mechanisms, with alterations linked to aging and neurodegenerative diseases. However, capturing fast physiological dynamics in preclinical models remains technically challenging due to high cardiac frequencies and small brain size. Here, we investigated the feasibility of zero echo time (ZTE) functional MRI (fMRI) to capture cardiac- and respiration-related pulsations across multiple temporal scales in the rat brain under isoflurane anesthesia. We used retrospective binning to assess cardiac- and respiration-related pulsations. Cardiovascular state was modulated with medetomidine, and functional connectivity analyses were performed to evaluate slower neural dynamics. ZTE fMRI robustly detected physiological pulsations across the brain, achieving effective temporal resolution of 8 ms, with the strongest signals observed in large arteries, consistent with an inflow‑based contrast mechanism. Cardiac pulsation amplitudes increased significantly under combined medetomidine–isoflurane anesthesia, whereas respiration‑related pulsations remained stable. Functional connectivity decreased under combined anesthesia, confirming ZTE fMRI sensitivity to slower neural dynamics. ZTE fMRI enables simultaneous assessment of cerebrovascular pulsatility and functional connectivity, providing a powerful tool for studying physiological brain dynamics in vivo.

## Introduction

Vascular pulsations are increasingly recognized as important markers of brain health and function. With each heartbeat, arterial walls expand and contract, driving the pulsatile flow of blood that delivers oxygen and nutrients to the brain. Following this, blood travels through the capillary network and is subsequently drained via the venous system. Beyond their role in cerebral perfusion, vascular pulsations have been implicated in brain clearance mechanisms. According to the glymphatic system theory,^1^ arterial pulsations serve as a driving force for the movement of cerebrospinal fluid (CSF) and interstitial fluid, facilitating the clearance of metabolic waste products from brain tissue. Disruptions or alterations in these pulsations—whether due to aging, vascular disease, or other pathological conditions—have been associated with impaired waste clearance, which may contribute to the development or progression of neurodegenerative disorders such as Alzheimer’s disease.^2^

Magnetic resonance imaging (MRI) is a well-established technique for assessing vascular structures and dynamics in the brain.^3^ Several MRI-based methods have been developed to image brain pulsations, glymphatic flow and vasculature, each with its advantages and limitations.^4,5^ One of the earliest and still widely used approaches involves the use of contrast agents to visualize CSF or blood movement (see van Osch et al.^6^ for review). However, the need for contrast agent injection into the CSF, safety concerns and possible adverse effects limit its applicability,^7^ particularly in cases where invasive procedures are not desirable or feasible.

An alternative approach relies on flow-sensitive sequences, such as phase-contrast MRI,^8^ which enables the quantification of velocity and flow patterns without the need for contrast agents. Balanced steady-state free precession (bSSFP) imaging is a method capable of providing information about flow dynamics,^9^ especially when combined with cardiac gating techniques. Many of the above-mentioned methods employ cine imaging, where data are retrospectively sorted according to the cardiac cycle to generate dynamic images of an “average” heartbeat. While effective for assessing periodic motion, this approach does not capture real-time fluctuations or transient events. In addition to flow-based techniques, MR elastography has been utilized to map brain tissue displacements,^10^ offering insights into the mechanical aspects of brain pulsations.

In humans, brain pulsations occur at relatively low frequencies, with a heart rate of around 1 Hz, making it feasible to capture pulsatile dynamics even with conventional MRI temporal resolutions.^11^ Recently, advanced methods such as magnetic resonance encephalography (MREG) have emerged, enabling whole-brain imaging with temporal resolution down to 100 ms,^12^ which makes it possible to image respiration- and heartbeat-related signals without aliasing. Such techniques, however, often require specialized hardware, such as multi-channel receiver arrays up to 64 channels, which are not readily available for preclinical MRI systems. Likewise, simultaneous multi‑slice (SMS) EPI substantially increases temporal sampling by exciting and acquiring multiple slices concurrently,^13^ allowing the acquisition of signals from major brain arteries and veins with high temporal resolution. However, effective SMS performance depends on high‑element coil arrays and parallel imaging reconstructions, which are generally lacking in preclinical MRI platforms.

In addition to the hardware-related limitations, preclinical imaging, particularly in rodents, poses other challenges, such as the high frequency of cardiac pulsations—several times faster than in humans—necessitating much faster imaging to resolve these dynamics. Furthermore, the significantly smaller brain size in rodents demands higher spatial resolution, compounding the complexity of capturing brain pulsations in vivo. Although challenging, the development of preclinical pulsation imaging techniques is indispensable for neuroscience research, as it enables controlled investigation of brain physiology and pathology, including pharmacological interventions and disease models in genetically modified animals. Such studies provide critical insights into cerebrovascular function, neurovascular coupling, and systemic influences on brain health, serving as a translational bridge to clinical applications.

Recently, ultrashort- and zero-echo time (zero-TE) MRI sequences have gained increasing attention for both structural and functional brain imaging.^14^ Zero-TE techniques offer several advantages over conventional sequences, particularly in terms of resilience to motion artifacts and magnetic field inhomogeneities,^15^ due to their high acquisition bandwidths and radial center-out *k*-space acquisition trajectories. Zero-TE sequences, in particular, are nearly silent during scanning, making them especially suitable for imaging studies in awake animals or sleep studies, where acoustic noise can be a significant confounding factor. Furthermore, the functional contrast in zero-TE is primarily driven by the inflow of fresh, unsaturated blood,^16^ making it naturally sensitive to vascular dynamics. Also, radial *k*-space sampling provides greater flexibility in designing acquisition schemes and enables retrospective reordering of the data according to physiological signals, such as cardiac or respiratory cycles.

In our previous fMRI work, we utilized a zero-TE variant, Multi-Band SWeep Imaging with Fourier Transformation (MB-SWIFT),^17^ to capture dynamic changes in the brain during repeated stimuli and recurring spontaneous events with high temporal resolution.^18^ In the present study, we extended this approach to explore physiological processes occurring throughout the cardiac and respiratory cycle using another zero-TE variant zero echo time (ZTE) sequence, which provides a shorter spoke repetition time (TR) than MB-SWIFT and thus improved sensitivity to fast physiological dynamics. To assess physiological modulation, we modulated the cardiovascular state of the animals using medetomidine anesthesia, which is known to alter heart rate and autonomic regulation. In addition, we evaluated functional connectivity using conventional analysis approaches to confirm that ZTE fMRI can reliably capture neural connectivity on slower timescales. Together, these aims tested the applicability of ZTE fMRI across multiple temporal scales in vivo.

## Materials and methods

### Animal experiments

All animal experiments were approved by the Finnish Animal Experiment Board (license ESAVI-19961-2023) and conducted in accordance with the European Commission Directive 2010/63/EU guidelines and the ARRIVE guidelines. Twelve adult Sprague–Dawley rats (27–37 weeks old, three male, 385–565 g, nine female, 240–324 g) were used for the experiments. Rats were group-housed and maintained on a 12/12 h light–dark cycle. Food and water were available ad libitum.

Animals were imaged at 9.4 T magnet interfaced with an Agilent DirectDRIVE console (Palo Alto, CA, USA) using a 22-mm loop surface local transmit/receive coil (Neos Biotec, Pamplona, Spain). The breathing rate of the animals was monitored using monitoring equipment (Model 1025; Small Animal Instruments, Inc., New York, NY, USA). Body temperature was measured using a rectal probe and maintained at 37° using a warm water circulation system (Corio CD; Julabo, Seelbach, Germany).

All rats underwent 36-min functional imaging under isoflurane anesthesia (5% induction, 2% maintenance in 30/70% O_2_/N_2_ carrier gas). In a subset of animals (8/12), a second imaging session was performed under combined medetomidine–isoflurane anesthesia to assess the effect of a change in physiological state on pulsations. To avoid potential sex-related effects, only female rats were included in the second measurement. Following the first imaging session, a medetomidine bolus (0.015 mg/kg, s.c.) was administered and the isoflurane concentration was gradually reduced to 0.5%. After a 20-min stabilization period required for heart rate to reach a steady state, a second functional imaging session was acquired using identical imaging parameters. At the end of the experiment, atipamezole (0.15 mg/kg, s.c.) was administered to reverse the effects of medetomidine. Functional data was acquired using ZTE sequence with following parameters: spoke TR 280 µs, 1947 spokes per imaging volume with a tennis ball^19^ trajectory, 4000 imaging volumes, field of view bandwidth 125 kHz, oversampling factor 2, 64 × 64 × 64 matrix size, 35 × 35 × 35 mm^3^ field of view, and flip angle 2.5°–3°.

Anatomical images were acquired with a magnetisation transfer (MT)—weighted MB-SWIFT sequence with the following parameters: 4000 spokes per spiral, 16 spirals, 64,000 spokes per imaging volume, spoke TR 3 ms, four radiofrequency pulses per spoke, field of view bandwidth 192 kHz, oversampling factor 2, 256 × 256 × 256 matrix size, 35 × 35 × 35 mm^3^ field of view, flip angle 6°. To increase anatomical contrast, a magnetization transfer pulse (γB1 = 125 Hz, offset 2000 Hz, duration of 20 ms) was applied every 32 spokes.

The electrocardiogram (ECG) was recorded simultaneously using Bittium NeurOne (Bittium Corporation, Oulu, Finland) differential amplifier system with 80 kHz sampling rate. Electrodes were placed under the skin to the sides of the animal, and the reference electrode was fixed on the back of the animal.

### ECG processing

As the ECG recordings were contaminated by MRI gradient artifacts, a denoising procedure was applied (Supplementary Figure 1). The approximate onset of each MRI volume was first estimated using trigger signals provided by the MRI console at the beginning of each volume acquisition. To refine the volume onset timing, cross-correlation with a gradient artifact template derived from several imaging volumes was performed on ECG data that were upsampled fivefold to an effective sampling rate of 400 kHz using linear interpolation. Upsampling was required because MRI data are acquired at a substantially higher sampling frequency than the original 80 kHz ECG recordings; without upsampling, the temporal precision of the template would be insufficient, potentially leading to incomplete removal of high-frequency gradient artifacts. Using the refined MRI volume onsets, a mean gradient artifact template was computed over a sliding window of 40 volumes and regressed from each corresponding volume in the ECG signal. The regressed data was downsampled to the original sampling rate. Finally, residual power line noise was removed using a 50 Hz notch filter.

After denoising the data, ECG peaks were detected using the MATLAB *findpeaks* function. After detecting the peaks and manually checking the quality of peak detection, the position of each peak in the MRI measurement (corresponding spoke and volume) was calculated based on MRI triggers.

### Respiration retrospective gating

**Figure 1.**
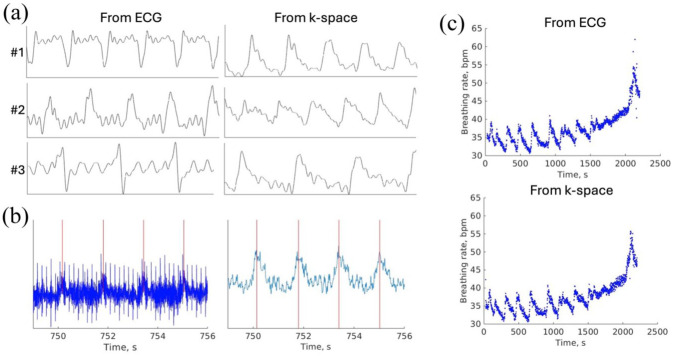
Detection of breathing peaks from the *k*-space data: (a) varying respiration waveforms in ECG complicate peak detection in the same phase for all animals (three different animals, 5-s window, lowpass filtered to 10 Hz). In contrast, respiration waveforms in *k*-space are more similar in shape, (b) ECG and *k*-space-derived time series with detected breathing peaks (red lines) for a representative animal, and (c) breathing rate from one fMRI measurement under isoflurane anesthesia detected from ECG and from *k*-space for the same animal.

Due to slightly varying electrode positions across animals, respiration waveforms derived from the ECG recordings differed in shape between subjects (Figure 1(a)), complicating the identification of breathing peaks corresponding to a consistent respiratory phase. We therefore extracted respiratory signals directly from the *k*-space data (Figure 1).

In radial ZTE acquisition, each spoke starts at the *k*-space center. Respiratory motion is predominantly a bulk geometric displacement of the imaged volume, and by the Fourier shift theorem, a spatial shift corresponds to a linear phase shift in *k*-space. The *k*-space center point captures this bulk displacement most directly, as it reflects the volume-integrated signal.^20^ We therefore used the phase of the first acquired *k*-space point after the dead-time gap as the respiratory surrogate signal. This point was chosen also because signal amplitude decays rapidly at positions further from the *k*-space center due to readout gradient-induced dephasing, making the first point after the dead-time gap the one with the highest signal-to-noise ratio.

Because different spoke directions sample different orientations in *k*-space, the phase of the first acquired point carries a spoke-direction-dependent offset unrelated to physiology. To remove this directional bias, we subtracted a mean volume template computed using a sliding window of 40 volumes, isolating the time-varying respiratory phase modulation from the static spoke-direction effect. The resulting phase time series was then low-pass filtered at 10 Hz to suppress high-frequency contributions, yielding a clean respiratory waveform from which breathing peaks were identified.

### fMRI data reconstruction

**Figure 2.**
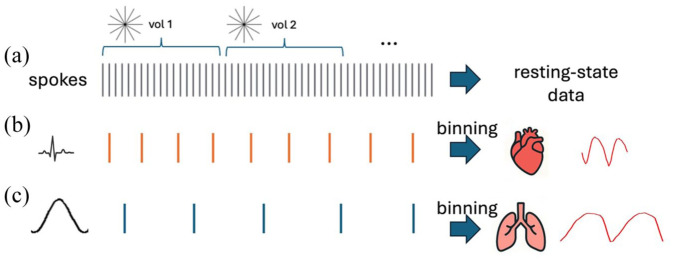
Different types of data reconstruction used in the study: (a) spokes were reconstructed into long resting-state time series without rebinning, (b) spokes were retrospectively binned according to heartbeats detected from simultaneous ECG measurements resulting in two heart cycles, and (c) spokes were retrospectively binned according to respiration peaks detected from the *k*-space center resulting in two breathing cycles.

The acquired ZTE data were reconstructed in three ways (Figure 2): (1) a standard reconstruction yielding 0.55 s temporal resolution for a series of 3D volumes, (2) retrospective binning with respect to heartbeat peaks detected from the ECG signal, enabling reconstruction of cardiac-phase-resolved images with subject-dependent 8–12 ms temporal resolution, and (3) retrospective binning with respect to respiration peaks detected from the *k*-space data, enabling reconstruction of respiration-phase-resolved images with 50–150 ms temporal resolution. In the binning approach, each spoke is assigned to a bin according to its position in the cardiac/respiratory cycle.^21^ Afterwards, all spokes assigned to a bin are reconstructed into a 3D volume, resulting in a series of 3D volumes, each representing one bin. In retrospective binning, *k*-space data are grouped retrospectively into temporal bins based on physiological timing rather than using prospective gating, allowing efficient use of the continuously acquired data and flexible reconstruction at multiple physiological timescales. Each R–R interval was individually divided into 20 equally spaced bins, so that each bin represents a fixed fractional phase of the cardiac cycle rather than a fixed absolute time duration. The same approach was applied to respiratory cycles. This per-cycle normalization inherently accommodates beat-to-beat and breath-to-breath variability, as each cycle is independently divided regardless of its duration. Note that the time axes in all cardiac- and respiration-gated plots represent bin index multiplied by the mean bin duration for the respective anesthesia condition; because individual cycle durations vary, these axes reflect the average temporal scale rather than a fixed time base.

For cardiac-gated pulsation profiles, time zero corresponds to the ECG-detected R-peak. For respiration-gated profiles, time zero corresponds to the detected peak of the *k*-space-derived respiratory waveform. In both cases, time zero represents a physiological reference point in the triggering signal and is not expected to coincide with the signal minimum or maximum in any given brain region. For cardiac pulsations, the delay between the R-peak and the local signal response depends on the vascular path length and pulse wave propagation time from the heart to each region. For respiratory pulsations, the phase relationship between the motion reference and regional brain signal changes varies with anatomical location and the specific mechanism coupling respiration to local signal fluctuations.

Within-acquisition heart rate and breathing rate stability were assessed in all animals that underwent each condition (*n* = 12 for isoflurane, *n* = 8 for combined anesthesia). For each animal and acquisition, stability was quantified as the coefficient of variation (CV) of the cycle duration (R–R interval for cardiac, peak-to-peak interval for respiratory cycles).

To quantify the consistency of pulsation waveform shapes, leave-one-out Pearson’s correlation coefficients were computed between each animal’s pulsation profile in the pterygopalatine artery and the mean profile of all remaining animals in the same anesthesia condition. For cross-condition comparison, each paired animal’s isoflurane waveform was correlated with its own combined anesthesia waveform. All shape correlations were computed as a function of fractional cycle phase (bin index) rather than absolute time.

In all three reconstruction approaches, after arranging spokes into volumes, the missing points in the center of the *k*-space were estimated using algebraic reconstruction.^22^ After that, phase correction was performed and images were reconstructed using iterative FISTA algorithm^23^ using three iterations. For the retrospectively binned data, all resulting 3D volume series were averaged, resulting in one 40-volume retrospectively binned 3D volume series per measurement.

### Coregistration

The anatomical image of one of the measured animals was chosen as a reference, and other anatomical images were coregistered to it. As the first step of coregistration, landmarks were drawn manually in all anatomical images, marking the position of lambda, azygos pericallosal artery (AZP), and left and right pterygopalatine arteries (PPA). Next, a rigid coregistration was performed between these landmarks. As the last step, non-linear SyN coregistration was performed using ANTs software (https://github.com/ANTsX/ANTs), using landmark-based rigid coregistration as the initial transformation.

Reconstructed functional images were slightly shifted to align with their corresponding anatomical image due to a half-voxel shift between MB-SWIFT and ZTE field of views. After that, transformation matrices from the anatomical coregistration were applied to each functional image.

### Analysis of retrospectively binned data

Independent component analysis (ICA) was used to extract major signal components from the group average functional data (MELODIC, FSL). Twenty components were extracted, and the main components were selected visually. Voxel-by-voxel correlation of group average data to each of the main component time series was performed to get spatial maps.

Regions of interest (ROIs) were drawn on top of the reference anatomical image to compare pulsations between anesthesia conditions. Main arteries visible on MT-weighted anatomical images were chosen for the analysis. In addition, ROIs were drawn in the superior sagittal sinus (SSS), aqueduct, somatosensory and motor cortices. Peak pulsation amplitudes were compared between anesthesia conditions using paired *t*-test (*p* < 0.05 considered significant, false discovered rate (FDR) corrected for multiple comparisons).

To assess the minimum amount of data needed for reliable pulsation detection, cardiac- and respiration-related binning was performed at both group and individual levels using progressively reduced dataset size. The resulting curves were correlated to the curve without data reduction.

### Analysis of resting-state data

To demonstrate that conventional resting-state functional connectivity can be extracted from the same ZTE fMRI data used for physiological pulsation mapping, group ICA (30 components) was performed on the resting-state data. Seven components, including two cortical, two subcortical, one ventricular, and two arterial components, were selected for further ROI analysis. Dynamic correlations between each pair of ROIs were computed using a sliding-window approach (250-volume window, 125-volume step) to account for the dynamic changes in the state of the brain during the long resting-state scan. Average correlation values were compared between anesthesia conditions using paired *t*-test with FDR correction for multiple comparisons.

## Results

Respiratory cycles were reliably extracted directly from the *k*-space data (Figure 1). In contrast to varying respiration waveforms in the ECG data, *k*-space-derived waveforms were similar across subjects (Figure 1(a)), allowing the determination of respiration peaks from the same phase of the respiratory cycle. The estimated breathing rates from *k*-space closely matched those derived from the ECG recordings (Figure 1(c)).

**Figure 3.**
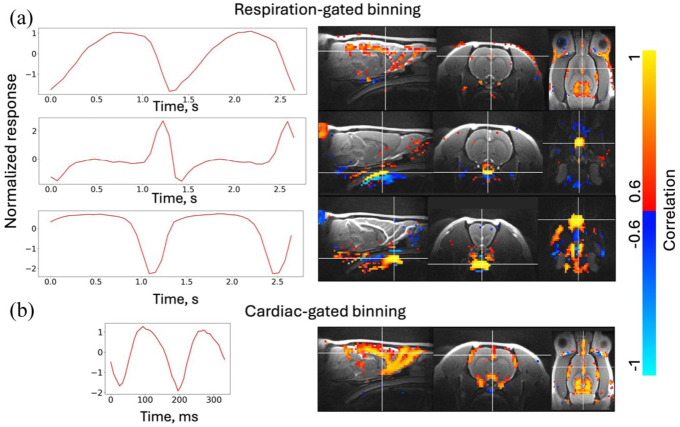
Group ICA components and correlation maps to identify vascular pulsations. The ICA components were detected with respiration-gated binning (a) and cardiac-gated binning (b). First component in respiration-gated binning is related to vascular pulsations, with second and third one being related to the movement of trachea and neck. In cardiac-gated binning, all components were related to vascular pulsations, with maps being highly similar (only one shown, additional maps can be found from Supplementary Figure 3). The *x*-axis represents the bin index multiplied by mean bin duration rather than the fixed time base.

Both cardiac- and respiration-related pulsations were detected in the rat brain (Figure 3). Cardiac and respiratory cycles were divided into 20 bins, so the effective resolution of cardiac-gated time series was 8–12 ms, and the effective resolution of respiration-gated time series was 50–150 ms. The respiration-related signal contained vascular pulsations, prominent in large arteries, as well as contributions from bulk motion adjacent to the trachea and near the neck. Cardiac-related pulsations were most prominent in large cerebral arteries, with ICA component time series peaking at different stages of the cardiac cycle (Supplementary Figure 2), likely representing the pulsation propagation wave at different stages.

**Figure 4.**
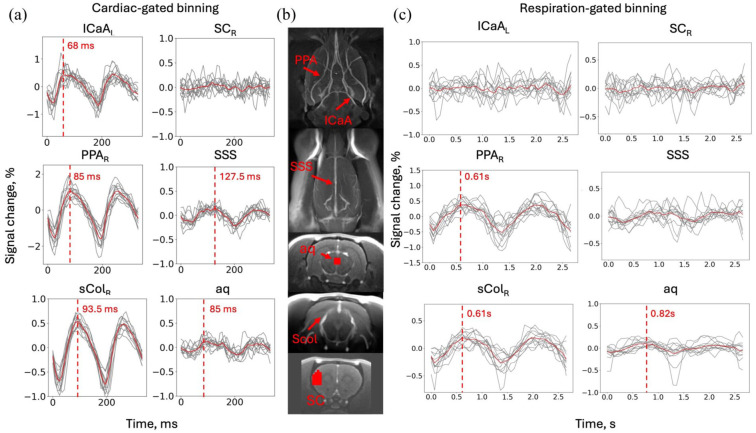
ROI analysis revealed different signals in major blood vessels in the brain. Red dashed lines mark where the curve maximum could be detected reliably in each ROI with timing of the peak indicated next to it. Red solid lines indicate average across subjects, while gray lines are subject-specific time series. For cardiac-gated pulsation profiles, time zero corresponds to the ECG-detected R-peak. For respiration-gated profiles, time zero corresponds to the detected peak of the *k*-space-derived respiratory waveform. The *x*-axis represents the bin index multiplied by mean bin duration rather than the fixed time base: (a) cardiac-gated binning, (b) ROIs used for the analysis, and (c) respiration-gated binning (*n* = 12). 3D views in (b) were created using Carimas software^24^ from the reference anatomical image. ICaA: internal carotid artery; PPA: pterygopalatine artery; sCol: supracollicular arterial network; SC: somatosensory cortex; SSS: superior sagittal sinus; aq: aqueduct; L: left side; R: right side.

Cardiac-gated pulsations differed in peak time and shape, depending on the region (Figure 4), with an earlier peak detected in the internal carotid artery (ICaA) and later in the pterygopalatine artery (PPA) and in the supracollicular arterial network (sCol), indicating propagation of the pulsation wave. Respiration-related pulsations were lower in amplitude and noisier, but also visible in the major arteries (Figure 4). In contrast, cortical gray matter regions exhibited weak or near-noise-level pulsations at both cardiac and respiratory frequencies. Weak pulsations were observed in the superior sagittal sinus (SSS) and aqueduct areas.

Heart rate remained stable within each acquisition under both anesthesia regimens. Under isoflurane alone (*N* = 12), the across-subject mean heart rate was 342.1 ± 27.4 bpm, with a within-measurement CV of 2.2% ± 0.85%. Under combined medetomidine–isoflurane anesthesia (*N* = 8), the mean heart rate was 266.9 ± 35.3 bpm with a within-measurement CV 2.8% ± 1.7%. Breathing rate also remained stable within each acquisition, although with greater physiological variability than cardiac rhythm, as expected. Under isoflurane alone, the mean breathing rate was 47.3 ± 7.0 breaths/min (CV: 7.6% ± 3.7%). Under combined anesthesia, the mean breathing rate was 33.4 ± 8.6 breaths/min (CV: 6.7% ± 1.8%). The higher respiratory CV compared with the cardiac CV is consistent with the known greater physiological variability of respiratory rhythm, even under stable anesthesia.

While absolute heart rates and breathing rates differed between animals, the shapes of cardiac-gated and respiration-gated pulsation profiles were highly consistent across animals within the same anesthesia regimen (Supplementary Figure 2). For cardiac-gated pulsations, within-condition leave-one-out correlations were high for both isoflurane (*r* = 0.93 ± 0.05, *n* = 12) and combined anesthesia (*r* = 0.96 ± 0.04, *n* = 8). Within-animal cross-condition correlations were also high (*r* = 0.89 ± 0.04, *n* = 8). For respiration-gated pulsations, within-condition correlations were moderate for both isoflurane (*r* = 0.70 ± 0.19, *n* = 12) and combined anesthesia (*r* = 0.64 ± 0.12, *n* = 8), with similar cross-condition correlations (*r* = 0.67 ± 0.12, *n* = 8). The subtle differences in cardiac and respiration waveform shapes between anesthesia conditions can be appreciated in Supplementary Figure 2.

**Figure 5.**
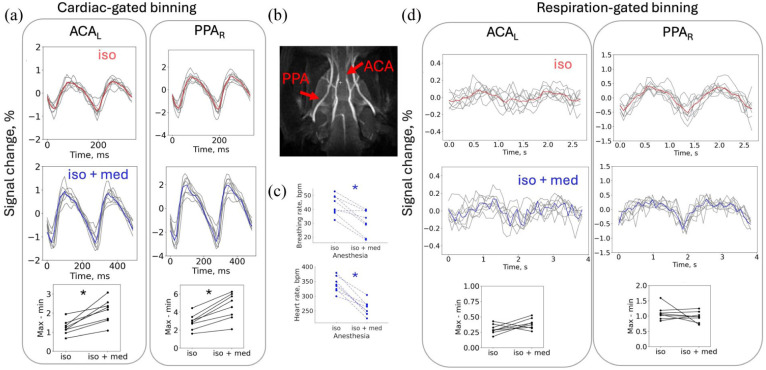
Effect of medetomidine anesthesia on the observed pulsations: (a) cardiac-gated binning showing increased pulsation amplitudes (gray: individual animals; red/blue: mean signals under isoflurane/isoflurane + medetomidine, *p < 0.01), (b) ROIs used for analysis, (c) breathing and heart rate decreased after medetomidine (*p < 0.01), and (d) with respiration-gated binning, amplitude changes were not significant (*p* > 0.05, *n* = 8). The *x*-axis represents the bin index multiplied by the within-regimen mean bin duration rather than the fixed time base. ACA: anterior cerebral artery; PPA: pterygopalatine artery.

Amplitudes of cardiac-related pulsations in major cerebral arteries were significantly higher under combined isoflurane and medetomidine anesthesia compared with isoflurane alone (*p* < 0.01, Figure 5). No significant differences in respiration-related pulsation amplitudes were observed between anesthesia conditions (*p* > 0.05).

**Figure 6.**
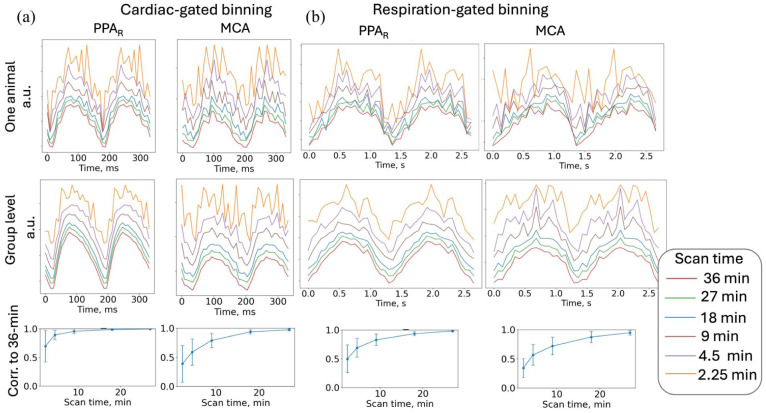
Data stability for different scan times: (a) data used for cardiac-gated binning and (b) data used for and respiration-gated binning. Time series from a representative animal (top) and group mean (middle) are shown for scan times from 36 to 2.25 min (4000–125 volumes). Curves are vertically offset for clarity. Bottom: correlation (mean ± SD) with the full 36-min acquisition, *n* = 12. The *x*-axis represents the bin index multiplied by mean bin duration rather than the fixed time base. MCA: middle cerebral artery; PPA: pterygopalatine artery.

The retrospective binning approach demonstrated robustness to substantial data reduction: even with an eightfold decrease in acquisition time (from 36 to 4.5 min), cardiac-binned pulsations in major arteries remained clearly detectable (Figure 6).

**Figure 7.**
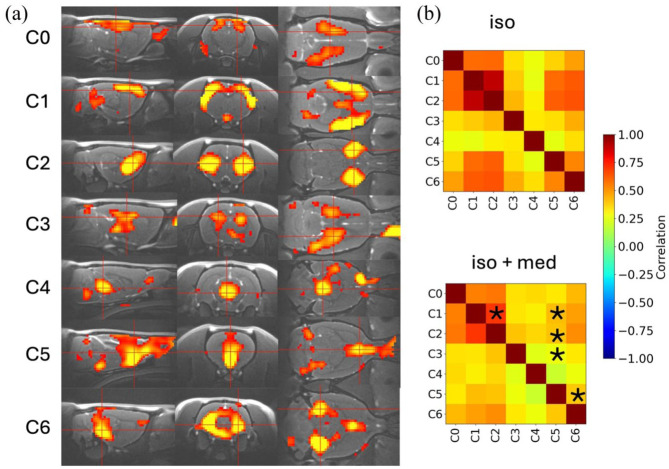
Resting-state analysis of fMRI data: (a) resting-state ICA components selected for the analysis and (b) correlation matrices between chosen components. Connectivities were lower under isoflurane-medetomidine combination (*p < 0.05, FDR-corrected for multiple comparisons), *n* = 8. C0 and C1: cortical components covering motor, visual, and somatosensory cortices; C2: caudate putamen; C3: hippocampus; C4: ventrical component, aqueduct; C5 and C6: arterial network.

Resting-state fMRI analyses revealed stable functional connectivity patterns and reproducible ICA components across conditions, including distinct cortical (C0, C1), subcortical (C2—caudate putamen, C3—hippocampus), ventricular (C4), and arterial (C5, C6) networks (Figure 7). Functional correlations between ICA components were significantly reduced under combined isoflurane and medetomidine anesthesia compared with isoflurane alone; specifically, connections between the cortical network and caudate putamen (C1–C2), the cortical network and an arterial component (C1–C5), caudate putamen and an arterial component (C2–C5), a hippocampal and an arterial component (C3–C5), and two arterial components (C5–C6) were significantly weaker under the combined regimen (Figure 7(b)). Notably, the arterial component C5 was involved in four of the five affected connections, indicating that medetomidine predominantly modulates correlations involving arterial pulsation networks.

## Discussion

This study demonstrates that ZTE fMRI can capture both cardiac- and respiration-related pulsations in the rat brain in 3D. Pulsations were most prominent in large cerebral arteries, consistent with the inflow-driven contrast mechanism of ZTE imaging.^16^

During the cardiac cycle, arterial wall expansion during systole facilitates inflow of unsaturated spins into the voxel, reducing the relative contribution of saturated stationary tissue and producing a signal increase. As the arterial diameter decreases and blood volume decreases, signal intensity correspondingly declines, which likely explains the strong arterial sensitivity and the comparatively weak pulsations observed in cortical gray matter, where blood volume changes are lower and larger volume fraction of static tissue dominates. In veins, blood has typically undergone multiple radiofrequency excitations and approaches steady-state magnetization, resulting in substantially reduced inflow contrast that may explain why venous pulsations were largely absent, except for modest signal fluctuations detected in the SSS. The origin of fluctuations in the SSS remains uncertain but may reflect partial volume effects with adjacent arteries or dynamic changes in venous blood volume and flow that are sufficient to generate a detectable inflow effect under certain conditions. Small but detectable signal fluctuations were observed within the ventricular system, including the cerebral aqueduct. They were substantially weaker than those observed in the major arteries and likely reflect a combination of subtle CSF motion, partial volume effects with adjacent vascular structures, and modulation of CSF flow driven by cardiac and respiratory cycles. In ZTE imaging, CSF is expected to exhibit near-steady-state magnetization due to its long T1 and comparatively slow flow, which limits the magnitude of inflow-related contrast. As a result, ventricular pulsations are expected to be more challenging to detect relative to arterial signals. Further work with CSF-specific encoding strategies will be required to disentangle true CSF pulsatility from partial volume and motion-related contributions.

Respiration-related pulsations were also detected throughout the brain, consistent with a previous study in humans.^25^ These signals likely arise from a combination of bulk motion, changes in intrathoracic pressure affecting cerebral blood flow, and respiration-modulated vascular pulsatility. Unlike cardiac pulsations, respiration-related amplitudes did not differ significantly between anesthesia conditions, suggesting that respiratory contributions are less sensitive to medetomidine-induced vascular changes.

We observed region-dependent differences in the timing of the cardiac-related signal peaks, which closely followed the anatomical organization of arterial inflow pathways. Arteries located near the entry points of blood into the imaging volume exhibited earlier signal peaks, consistent with more rapid arrival of unsaturated blood. These findings underscore the sensitivity of ZTE-based pulsation mapping to vascular anatomy and flow pathways.

In this study, we used a low FA (2.5°–3°) and short spoke TR (280 µs), resulting in relatively mild saturation of stationary tissue. Under these conditions, inflow contrast is expected to be most pronounced in large arteries, where flow velocities are high and unsaturated blood is continuously replenished. As blood propagates further into the distal vasculature, it undergoes progressively more RF excitations and approaches steady-state magnetization, reducing the inflow contrast, which is consistent with our observation of strong pulsations in major arteries and comparatively weak pulsations in cortical parenchyma and veins. Higher FAs or shorter TRs would increase the saturation of stationary tissue and thereby amplify the inflow contrast, but would also cause inflowing blood to saturate more rapidly as it travels downstream, potentially confining detectable pulsations to the most proximal arterial segments. Conversely, lower FAs or longer TRs would reduce stationary tissue saturation but allow unsaturated magnetization to propagate further into the vascular network before reaching steady state, potentially extending sensitivity to smaller or more distal vessels at the cost of reduced overall contrast. This parameter dependence suggests that ZTE fMRI could in principle be tuned to probe different vascular compartments, although systematic optimization was beyond the scope of this study.

While inflow is likely the dominant contrast mechanism with our imaging parameters, other contributions cannot be entirely excluded. Pulsatile changes in cerebral blood volume (CBV) alter the fractional composition of tissue and blood within a voxel, which could modulate signal intensity independent of inflow effects. Recent preliminary work from Mackinnon et al.^26^ modeled the contributions to SORDINO fMRI contrast in the rodent brain at 9.4 T using head-only RF excitation. They explored contrast from inflow-enhanced CBV, where increased vascular-space-occupancy of unsaturated inflowing blood produces a positive signal change, and alterations in tissue oxygen levels, which modulate T1 relaxation rates and contribute to signal changes of a similar order of magnitude as CBV. In another preliminary work available as preprint, Özen et al.^27^ reported analogous considerations for ZTE fMRI in humans, noting that the relative weighting of these mechanisms depends on sequence parameters and coil configuration, and that whole-body RF excitation shifts the dominant contrast toward T1-mediated oxygenation effects. In our experimental setup, several factors favor inflow as the primary contrast source. We used a local transmit/receive surface coil, which produces a spatially confined excitation profile: blood entering the sensitive volume has experienced few prior RF excitations and retains a substantial magnetization advantage over saturated stationary tissue. Furthermore, the strong pulsatile signals we observe are overwhelmingly localized to major arteries, with comparatively weak fluctuations in parenchyma and veins—a pattern most consistent with inflow-driven contrast. Nevertheless, T1-mediated oxygenation and CBV-related partial volume effects may contribute to the signal fluctuations we observe, and disentangling these from pure inflow contrast would require systematic variation of sequence parameters or complementary measurements. Future studies employing multiple FA/TR combinations and different coil configurations could help characterize the relative contributions of these mechanisms across vascular and fluid compartments.

Administration of medetomidine in combination with isoflurane had a pronounced effect on cardiac-related pulsations, resulting in significantly increased pulsation amplitudes in major cerebral arteries and altered temporal characteristics of the cardiac waveform. These effects were widespread, indicating a global vascular response. Medetomidine is a potent α2-adrenergic agonist that increases vascular tone and arterial pressure,^28^ whereas isoflurane induces vasodilation and increases cerebral blood flow.^29^ The observed enhancement of arterial pulsatility under combined anesthesia is consistent with previous reports demonstrating increased vascular resistance and arterial pressure following medetomidine administration.^30^ The combined anesthetic regimen is often considered to yield a more physiologically balanced vascular state, mitigating excessive vasodilation while maintaining adequate perfusion.^31^

Increased arterial pulsatility under medetomidine may have implications beyond vascular dynamics alone. Previous work has shown that medetomidine–isoflurane anesthesia enhances glymphatic solute transport in rats compared with isoflurane alone.^32^ Although the present study did not directly assess glymphatic flow, the observed increase in cardiac-driven pulsations may contribute to more efficient perivascular fluid transport, warranting further investigation.

An important consideration for retrospective cardiac binning is whether variations in heart rate affect the resulting pulsation profiles. In our approach, each cardiac cycle is independently divided into 20 fractional phase bins, meaning that the binning inherently normalizes for differences in cycle duration. Within each acquisition, heart rate was highly stable (CV: 2.2% ± 0.85% under isoflurane, and 2.8% ± 1.7% under combined anesthesia), confirming that the binned data represent a consistent physiological state rather than an average over widely varying cycle durations. Breathing rate showed somewhat greater variability (CV: 7.6% ± 3.7% under isoflurane, and 6.7% ± 1.8% under combined anesthesia), consistent with the known greater physiological fluctuation of respiratory rhythm.

Within each anesthesia condition, cardiac-gated and respiration-gated pulsation waveforms were highly and moderately consistent across animals, respectively (Supplementary Figure 2), suggesting that the temporal profile of the pulsatile response is governed primarily by vascular architecture and pulse wave propagation rather than by the specific physiological rate.

Cardiac-gated and respiration-gated pulsation waveform shapes were also highly and moderately consistent, respectively, across anesthesia conditions, indicating that the overall waveform morphologies are largely preserved even across regimens. Subtle differences in shapes can still be noted across regimens (Supplementary Figure 2), but their interpretation requires caution. Because each R–R interval is independently divided into fractional phase bins, the same bin index corresponds to different absolute times in the two conditions. Furthermore, the relationship between heart rate and systolic duration is nonlinear—systole is relatively preserved while diastole shortens at higher heart rates—meaning that the temporal structure of the cardiac cycle is not simply scaled between conditions. Consequently, observed differences in waveform shape between isoflurane and combined medetomidine-isoflurane anesthesia may reflect not only pharmacological changes in vascular tone and compliance but also the altered temporal partitioning of systole and diastole at different heart rates. Disentangling these contributions would require systematic variation of heart rate within a fixed anesthesia protocol, which was beyond the scope of this study. We also note that the heart rate range in our study was limited to that observed under two controlled anesthesia regimens, and more extreme variations in heart rate could in principle alter pulsation characteristics through changes in cardiac output, stroke volume, or vascular compliance.

Resting-state analyses of normally reconstructed resting-state data revealed stable and reproducible ICA components across anesthesia conditions, including distinct cortical, subcortical, ventricular, and arterial networks, indicating that connectivity and pulsation information can be extracted from the same dataset. This dual utility is important because it means that a single ZTE fMRI acquisition can serve both physiological monitoring and conventional connectivity analyses, reducing total scan time and avoiding the need for separate acquisitions. Functional correlations between components were significantly reduced under combined medetomidine–isoflurane anesthesia compared with isoflurane alone. Strikingly, four of the five affected connections involved the arterial component C5, including its correlations with the cortical network (C1), caudate putamen (C2), hippocampus (C3), and another arterial component (C6). This pattern suggests that medetomidine does not uniformly suppress all functional correlations but rather preferentially disrupts coupling between arterial pulsation networks and other brain components. This is consistent with the known vasoconstrictive properties of medetomidine mediated through α2-adrenergic receptor agonism,^28^ which would be expected to alter arterial pulsatility and its coupling to neural and cerebrospinal fluid dynamics. The single affected non-arterial connection—between the cortical network and caudate putamen (C1–C2)—is consistent with previous reports of medetomidine-induced suppression of cortico-subcortical functional connectivity^33^ and highlights the importance of accounting for anesthesia state when interpreting resting-state fMRI metrics alongside physiological pulsations.

Although the original measurement took 36 min, we showed that similar information could be extracted from a much shorter measurement. Importantly, even with an eightfold reduction in acquisition time, cardiac pulsations in major arteries remained clearly detectable (correlation to 36-min curve >0.5), highlighting the efficiency and sensitivity of the method. Potentially, more advanced data analysis approaches could reduce the measurement time even further, which could enable detection of minute-scale changes in pulsation parameters.

One of the key practical advantages of this framework is that it does not require specialized hardware beyond standard physiological monitoring. Moreover, both cardiac and respiratory signals were directly observable in the raw *k*-space data, particularly near the center of *k*-space, reflecting the fact that ZTE samples low spatial frequencies that are highly sensitive to global physiological fluctuations with high temporal resolution. The principle of extracting physiological motion signals directly from *k*-space data has been established in the context of cardiac MRI, where self-gating approaches using the *k*-space center have been employed for retrospective cardiac gating for over two decades.^34^ Similar strategies have been applied to respiratory motion in body imaging.^20,35^ Our work extends this concept to brain imaging with ZTE fMRI, where we demonstrate that respiratory peaks can be reliably extracted from the phase of the *k*-space center point, providing a robust alternative to external physiological recordings. With further development, ZTE has the potential to extract physiological waveforms directly from imaging data, allowing fully self-navigated, ECG-free acquisitions.

Because retrospective binning is performed in *k*-space, motion must be considered prior to cardiac or respiratory sorting. In the present study, anesthesia minimized animal motion, so explicit correction was not needed. For applications involving awake animals or increased motion, rigid-body motion correction can be implemented directly in *k*-space, including translational and rotational adjustments.^36^

A few limitations should be noted. Retrospective binning inherently requires averaging over multiple physiological cycles, precluding detection of transient or non-periodic events. Real-time or single-cycle imaging approaches would be necessary to capture such dynamics. Additionally, medetomidine experiments were conducted exclusively in female rats to reduce variability and avoid confounding sex-dependent vascular effects. Future studies should evaluate whether the observed effects generalize to male animals.

## Conclusion

In this study, we demonstrated that ZTE fMRI can effectively capture cardiac- and respiration-related brain pulsations in vivo in rats, including the spatiotemporal propagation of pulsation waves across the cerebrovascular network. The strongest pulsations were observed in large arteries, consistent with the inflow-based contrast mechanism of ZTE. The method was sensitive to pharmacologically induced physiological changes, revealing increased cardiac-related pulsation amplitudes under combined medetomidine-isoflurane anesthesia compared with isoflurane alone, while respiration-related pulsations were largely unaffected. At the same time, ZTE fMRI allowed measurement of functional connectivity patterns comparable to those reported with conventional fMRI, indicating that the method captures both fast physiological dynamics and slower functional fluctuations. Importantly, these capabilities were achieved without the need for specialized hardware, highlighting ZTE fMRI as a practical tool for investigating cerebrovascular pulsatility and brain dynamics across multiple timescales.

## Supplemental Material

sj-docx-1-jcb-10.1177_0271678X261445230 – Supplemental material for Mapping cardiac and respiratory pulsations simultaneously with functional connectivity in the rat brain using zero echo time fMRI Supplemental material, sj-docx-1-jcb-10.1177_0271678X261445230 for Mapping cardiac and respiratory pulsations simultaneously with functional connectivity in the rat brain using zero echo time fMRI by Ekaterina Paasonen, Petteri Stenroos, Antonio Caulin Atienzar, Juhani Utriainen, Raimo A Salo, Mikko Kettunen, Sara Ponticorvo, Shalom Michaeli, Silvia Mangia, Jaakko Paasonen and Olli Gröhn in Journal of Cerebral Blood Flow & Metabolism
