# Supplementary material for: Mapping cardiac and respiratory pulsations simultaneously with functional connectivity in the rat brain using zero echo time fMRI
Source: J Cereb Blood Flow Metab. 2026 May 6:0271678X261445230. Online ahead of print. doi: 10.1177/0271678X261445230 (PMC13152982; doi:10.1177/0271678X261445230)
Supplement: sj-docx-1-jcb-10.1177_0271678X261445230 – Supplemental material for Mapping cardiac and respiratory pulsations simultaneously with functional connectivity in the rat brain using zero echo time fMRI [file sj-docx-1-jcb-10.1177_0271678X261445230.docx]

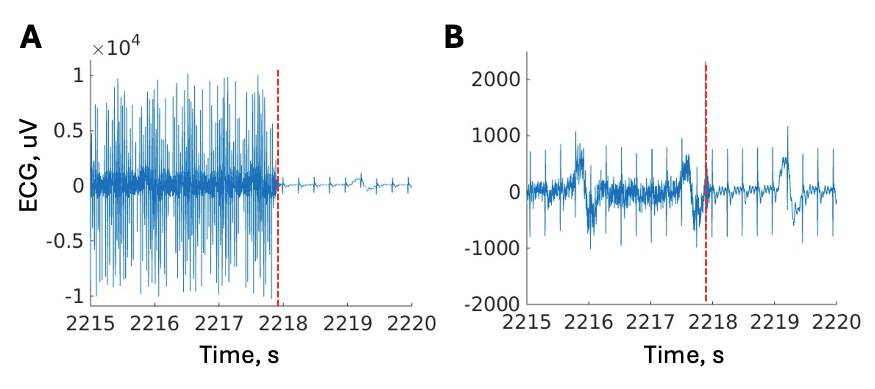


**Supplementary Figure 1.** ECG quality before (A) and after (B) MRI artifact removal from a representative animal. Red line marks end of the ZTE measurement.


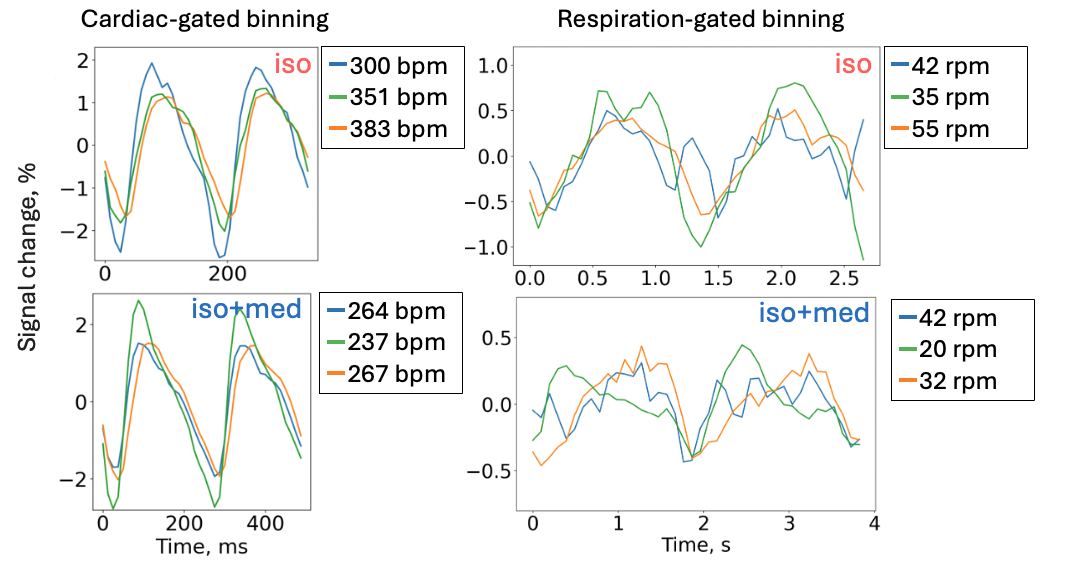


**Supplementary Figure 2**. Example of cardiac-gated and respiration-gated pulsation shapes consistency in pterygopalatine artery under isoflurane and isoflurane/medetomidine combined anesthesia in three animals with different heart and respiration rates. Rather than a fixed time base, the x-axis represents the bin index multiplied by the within-regimen mean bin duration.


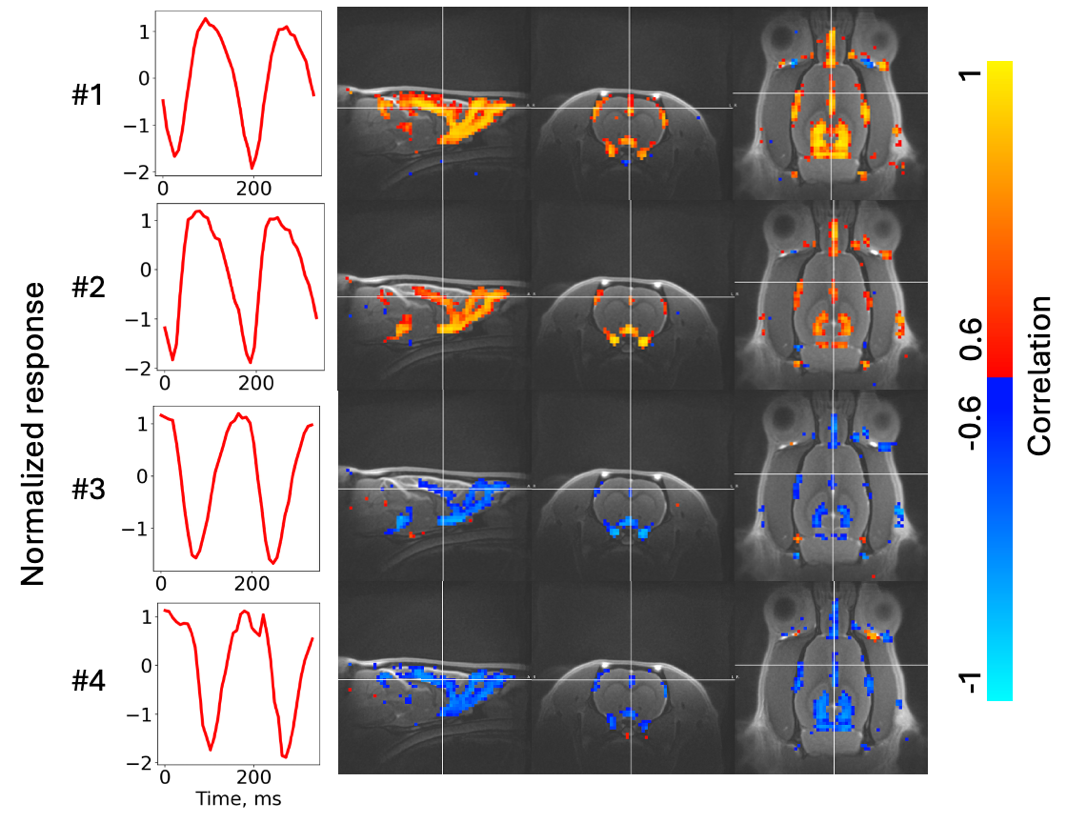


**Supplementary Figure 3.** Vascular group ICA components time series and correlation maps detected with cardiac-gated binning.
